# Air pollution: 6.6 million premature deaths in 2050!

**DOI:** 10.1007/s12471-015-0763-9

**Published:** 2015-10-21

**Authors:** E.E. van der Wall

**Affiliations:** Netherlands Society of Cardiology/Holland Heart House, Moreelsepark 1, 3511 EP Utrecht, The Netherlands

The theme air pollution has gained increased attention over the past decade [1, 2]. Recent research indicates that outdoor air pollution leads to more than 3 million premature deaths around the world each year. These impressive findings were published on 16 September 2015 in Nature by Lelieveld et al. from the Max Planck Institute for Chemistry, Atmospheric Chemistry Department, Mainz, Germany [3]. The authors investigated worldwide the link between premature mortality and seven sources of atmospheric pollutants in urban and rural environments. China showed the most premature deaths with 1.36 million deaths, with India ranking second at 645,000 deaths and Pakistan third with 111,000 deaths. Approximately 75 % of the deaths are due to cerebral strokes and heart attacks. The premature mortality can be linked to a wide range of causes including the effect on human health of outdoor air pollutants such as ozone and fine particulate matter with a diameter smaller than 2.5 µm (PM2.5). Air pollutants induce inflammation and increase oxidative stress within vascular tissue, ultimately leading to endothelial dysfunction, atherosclerosis, myocardial infarction, heart failure, cardiac arrhythmias, and cerebral strokes. In particular low-quality fuels used for cooking, heating and waste disposal have resulted in a high number of premature deaths in densely populated parts of Asia, including China, India, Bangladesh, Indonesia and Nepal. Agricultural emissions were the leading cause of premature air pollution deaths in the Eastern United States, Europe, Russia, Turkey, Korea and Japan, followed by traffic and power generation emissions. Model projections based on a business-as-usual emission scenario indicate that the contribution of outdoor air pollution to premature mortality could double by 2050. The authors of the Nature paper foresee therefore that, if no further interventions are undertaken, the annual toll from polluted air may lead to 6.6 million premature deaths by 2050, with the biggest increase in Asia.

Also at the European level the effects of air pollution have obtained increased consideration over the past years. In the European Study of Cohorts for Air Pollution Effects (ESCAPE) network of 22 European cohort studies (> 300,000 subjects), PM2.5 effects on cardiovascular mortality were approximately twofold larger than the previous estimates, and associations persisted even among subjects with residential PM2.5 mean annual concentrations below 15 µg/m^3^ [4]. In January 2015, the European Heart Journal (EHJ) published an Expert Position Paper on *Air Pollution and Cardiovascular Disease* [5]. Based on this paper, the European Society of Cardiology (ESC) declared air pollution to be an additional risk factor for cardiovascular disease, independent of the other well-known major risk factors [6–10]. At the 2015 annual ESC Congress in London, 29 August–2 September, the spotlight of the congress was Environment and the Heart. Emphasis was laid on exploring how environmental risks, in particular air and noise pollution, substantially impact on cardiovascular health. The discussions at the ESC Congress made it very clear that there should be more awareness about the need to create a healthy environment to protect heart health, and more efforts made to encourage policymakers to take action. In response to this, the ESC has joined forces with the European Heart Network (EHN), and the European Association for Cardiovascular Prevention and Rehabilitation (EACPR), to launch a petition asking European Union (EU) policy makers to show commitment and act accordingly.

At our national level, the group of Brunekreef et al. from the Netherlands Julius Center for Health Sciences and Primary Care, University Medical Center, Utrecht, has acquired world-wide recognition for performing pivotal studies addressing the effects of air pollution on human health [11–13]. Brunekreef et al. [13] showed convincingly that traffic-related air pollution (*Cave Volkswagen Diesel, EEvdW*) and several traffic exposure variables in the Netherlands are associated with increased mortality. Interestingly and along those lines, at the end of September 2015 it was announced, through the public media by the National Institute for Public Health and the Environment (RIVM), that the air at and around our National Airport Schiphol is polluted with high concentrations of ultrafine particles (less than 0.1 µm in diameter = 100 nm) [14]. At peak moments 300,000 particles/cm^3^ have been measured. Air pollution is mainly due to take-off and landing of airplanes and can still be detected within a radius of 15 km of the airport. The detrimental effects on human health and the environment have yet to be determined. If so, the question could arise whether Schiphol has to move to the North Sea(?).

To summarise, there is now abundant evidence that air pollution contributes to the risk of cardiovascular disease and associated mortality. Air pollution should be viewed as one of several major modifiable risk factors in the prevention and management of cardiovascular disease [15]. Further research should explore the optimal methods of air pollution reduction and document its effects on the incidence of cardiovascular disease and related mortality in order to force policy makers worldwide to intensify the efforts required for effective legislation on sincere reduction of air pollution.
